# The dysregulation of the hypothalamic–pituitary–adrenal axis in diet-induced prediabetic male Sprague Dawley rats

**DOI:** 10.1186/s12986-020-00532-1

**Published:** 2020-12-11

**Authors:** Palesa Mosili, Bongeka Cassandra Mkhize, Phikelelani Ngubane, Ntethelelo Sibiya, Andile Khathi

**Affiliations:** 1 Department of Human Physiology, School of Laboratory Medicine and Medical Sciences, College of Health Sciences, University of KwaZulu-Natal, Room E2-401, Westville, 4000 South Africa; 2 Pharmacology Division, Faculty of Pharmacy, Rhodes University, Grahamstown, 6140 South Africa

**Keywords:** Prediabetes, High-fat high-carbohydrate diet, Hypothalamic–pituitary–adrenal axis, Cortisol, Corticosterone, Stress

## Abstract

**Background:**

Altered function of the hypothalamic–pituitary–adrenal (HPA) axis in type 2 diabetic patients, a condition preceded by pre-diabetes, has been shown to increase the risk of depression as well as cause downstream effects resulting in upregulation of gluconeogenesis and dyslipidemia. In addition, stress, either psychological from managing diabetes or lifestyle related, further activates the HPA axis causing an exaggerated stress response. This study investigated the activity of the HPA axis in selected markers of glucose handling, and the stress response relative to components of the HPA axis in a diet-induced pre-diabetic rat model.

**Methods:**

Sprague Dawley Rats were randomly divided into non-pre-diabetic group (NPD) and pre-diabetic group (PD) (n = 6, per group) over a 20-week induction period and a further 12-week experimental period to get 32 weeks. At the end of the 20 and 32-week periods, glucose handling using the Homeostasis Model Assessment indices, adrenocorticotropic (ACTH) and corticosterone (CORT) concentrations were measured. Stress was induced and the forced swim test were performed in the 12-week experimental week. At the end of 32 weeks glucocorticoid and mineralocorticoid hippocampal receptors were also measured.

**Results:**

Impaired glucose handling in the PD group as well as increase in corticosterone was observed at the end of both 20 and 32-week periods by comparison to NPD groups. No changes were observed in ACTH concentration at week 20 while, at week 32, a decrease in plasma ACTH concentration was observed in the PD group by comparison to the NPD group. The stressed-induced animals were stressed using the forced swim test: the behaviour observed showed an increase in immobility time in the PD stressed group by comparison to the NPD group. This was followed by the observation of a decrease in ACTH and CORT concentration in the PD stressed group by comparison to the NPD stressed group. Mineralocorticoid and glucocorticoid receptors gene expression were elevated in the stressed PD group relative to the stressed NPD group.

**Conclusion:**

These observations, together, suggest that diet-induced pre-diabetes is associated with impaired HPA axis activity and deteriorating response to stress.

## Background

Pre-diabetes is defined as an intermediary state of hyperglycaemia that occurs between normoglycaemia and type 2 diabetes mellitus (T2DM) with blood glucose concentrations above normal but below the threshold for diagnosis of diabetes [1, 2]. It is further characterised by reduced insulin sensitivity in the peripheral tissues such as skeletal muscles [1]. Increased consumption of high caloric diet and physical inactivity coincided with the increased prevalence of diabetes and pre-diabetes [3, 4]. In 2012, the Centre for Diabetes and Endocrinology South Africa reported that three and a half million South Africans suffered from diabetes and estimated that five million people in South Africa have pre-diabetes [4]. Globally, the International Diabetes Federation (IDF) estimated that by 2045, 629 million are expected to have T2DM [3]. While T2DM is often associated with macro-and microvascular complications, studies indicate that poor management of everyday stress in diabetic patients is associated with increased risk of depression and anxiety [5–7]. This is said to be due to the constant activation of the hypothalamic–pituitary–adrenal (HPA) axis observed in type 2 diabetic patients [8].

The hypothalamic–pituitary–adrenal (HPA) axis plays a role in glucose homeostasis during acute stressful conditions [9–11]. Glucocorticoids, cortisol in humans or corticosterone in rodents, the end product of the HPA axis, is a catabolic hormone with the primary function of increasing availability of energy during a stressed condition by having an effect on the metabolism of carbohydrate and lipids [12, 13]. However, dysregulation of the HPA axis in T2DM has been shown to further worsen the hyperglycaemic state causing hyperlipidemia and dyslipidemia as well as increase the risk of depression in diabetic patients [8, 13–16]. The prevalence of depression and anxiety is much higher in diabetic patients than it is for non-diabetic individuals [17, 18]. Several studies attribute this to stress associated with the diagnosis and management of diabetes as well as treatment of the disease [19, 20]. The increased stress activates an already heightened HPA axis causing an elevated stress response resulting in the development of a behavioural response that includes decreased physical exercise and increased consumption of unhealthy diet. [5, 20–22]. The consumption of a more palatable food rich in refined carbohydrates and saturated fats as a coping mechanism may dampen the stress response by triggering the release of dopamine which activates the reward pathways found in the brain triggering the release of serotonin [23, 24]. The release of serotonin towards the hypothalamus overrides satiety and hunger pathways resulting in either further consumption or diminished intake of food [23]. The constant release of serotonin due to persistent consumption of this diet exacerbates the diabetic conditions resulting in further activation of the HPA axis [6, 24–26]. The persistent activation of the HPA axis may lead to dysregulation of the HPA axis resulting in depressive symptoms such as psychomotor retardation as seen in some diabetic patients [22, 27]. However, it is unknown whether these changes are present in a prediabetic state.

Previous studies in our laboratory developed a high fat high carbohydrate (HFHC) diet-induced prediabetic animal model which mimics the human condition of pre-diabetes [28, 29]. Several studied have revealed that various complications seen in T2DM begin in the prediabetic state [28–30]. However, the effect of pre-diabetes on the HPA axis and stress response in the prediabetic animal model has not yet been investigated. Hence, the aim of this study was to investigate HPA axis function and the stress response in a diet-induced prediabetic rat model.

## Methods

### Animals and housing

Male Sprague–Dawley rats (150–180 g) used in this study were bred and housed in the Biomedical Research Unit (BRU) of the University of KwaZulu-Natal. All animal procedures and housing conditions were approved by the Animal Research Ethics Committee of the University of KwaZulu-Natal, which conforms to the principles and guidelines of Canadian Council on Animal Care (ethics no: AREC/024/018D). The animals were maintained under standard laboratory conditions of constant temperature (22 ± 2 °C), carbon dioxide (CO_2_) content (< 5000 p.m.), relative humidity (55 ± 5%) and illumination (12 h light/dark cycle, lights on at 07h00). The noise level was maintained at less than 65 decibels approved. The animals were allowed access to food and fluids ad libitum. The animals acclimatized to their new environment for 1 week while consuming standard rat chow and tap water before the induction of pre-diabetes by exposure to a well-established experimental diet (HFHC) [29].

### Induction of pre-diabetes mellitus

Sprague–Dawley rats were randomly assigned to two diet groups, group A and B (n = 18 per group): Experimental pre-diabetes was induced in male Sprague–Dawley rats using a protocol previously described by Luvuno et al. [29]. In summation, the group B animals were exposed to HFHC diet supplemented with 15% fructose for 20 weeks and designated as the prediabetic group (PD) while group A which was designated non-prediabetic group (NPD) was exposed to standard rat chow with normal drinking water for the equal number of weeks. After 20 weeks, the American Diabetes Association criteria for the diagnosis of pre-diabetes was used to diagnose all animals for pre-diabetes, i.e. animals that had prediabetic signs including fasting blood glucose concentrations of 5.6–7.1 mmol/L, oral glucose tolerance test (OGTT) 2-h glucose concentration of 7.1–11.1 mmol/L and plasma triglycerides concentration of greater than 2 mmol/L were considered prediabetic and all above measurements below the prediabetic threshold were considered non-prediabetic. At the end of 20 weeks, 6 rats from each group (n = 6 per group) were sacrificed for biochemical analysis. This was considered phase 1 of the study. The rest of the rats continued with their respective diets for 12 weeks more which would be regarded as phase 2 of the study where food intake was monitored, and stress-induced to complete 32 weeks to allow further development of pre-diabetes.

### The Homeostasis Model assessment

At the end of both phases, the Homeostasis Model assessment was used to measure HOMA-IR, HOMA-S and HOMA-β indices to assess insulin resistance, insulin sensitivity and beta-cell function capacity were calculated using the HOMA2 Calculator v2.2.3 program [31]. In homeostasis model assessment (HOMA) insulin resistance is expressed as HOMA-IR value < 1.0 = insulin-sensitive, > 1.9 = early insulin resistance, > 2.9 = significant insulin resistance. Insulin sensitivity is expressed as HOMA-S% where the higher the percentage the higher the insulin sensitivity of the subject. Beta-cell secretory capacity is expressed as HOMA-β% where the higher the value the more the beta-cells secrete more insulin to handle existing blood glucose level [32].

### Experimental procedures

#### Unpredictable chronic mild stress

At the beginning of week 32, the animals were further randomly divided into four groups according to rats that would be stressed. The NPD group was split into two groups (n = 6 per group) which were designated non-prediabetic non-stressed (NPD-NS), and non-prediabetic stressed (NPD-S) groups. The PD group was divided into two groups (n = 6 per group) as well and were designated as prediabetic non-stressed (PD-NS), and prediabetic stressed (PD-S). The stressed rats experienced unpredictable chronic mild stress (UCMS) which according to literature may last for 5 days or more compared to acute stress which has been shown to be 1 to 3 days depending on the type of stressor and duration of the stressor [33, 34]. However, for the purpose of this study, 5 days of UCMS was conducted using the physical restraint stress (3 days) and forced swim test (2 days).

### Physical restraint stress

Non-prediabetic stressed (NPD-S), and prediabetic stressed (PD-S) rats were placed in a clear cylindrical tube with ventilation holes for 30 min in an isolated room between 09h00 and 10h00 for 2 days prior to the forced swim test and again for an additional day after forced swim test.

### Forced swim test

During week 31 on day 5, 2 days after the physical restraint, the forced swim test was performed. Clear or transparent PVC 50-cm high, 22 cm diameter cylindrical tanks were filled with tap water (23 ± 2 °C) to a water depth adjusted according to the rat’s size so that the rat’s tail could not touch the bottom of the container. Water was replaced after each session. Forced swim sessions were carried out and video recorded in the BRU behavioural room. Each rat underwent two sessions, the pre-test stage and 24 h later, the test stage. During the pre-test session, the rats were individually placed in the water for a 15-min forced swim session. During the test session, 24 h later, the rats were placed in the water again for 5 min. Immediately after each session, rats were dried with a cloth towel for 10–20 s and placed back in their home-cages. The following behavioural measures were scored by the researcher during the first 5-min period of each session: (1) Swimming: The rat shows smooth, coordinated movements with forelimbs or hind limbs moving in a paddling fashion and the water surface is not broken by the limbs. (2) Climbing/Struggling: The rat shows strong, vigorous movements with the forelimbs and hind limbs moving forcefully to break the water surface vigorously. (3) Immobility: The rat floats or only makes basic movements to keep the nose above the water surface. To record only the full presentation of behaviours, a behaviour began to be recorded after it lasted 2 s.

## Blood collection and tissue harvesting

For blood collection, all animals were anaesthetised with Isofor (100 mg/kg) (Safeline Pharmaceuticals (Pty) Ltd, Roodeport, South Africa) via a gas anaesthetic chamber (Biomedical Resource Unit, UKZN, Durban, South Africa) for 3 min. While rats were unconscious, blood was collected by cardiac puncture and then injected into individual pre-cooled heparinized containers. The blood was then centrifuged (Eppendorf centrifuge 5403, Germany) at 4 °C, 503 g for 15 min. Plasma was collected and stored at − 70 °C in a Bio Ultra freezer (Snijers Scientific, Holland) until ready for biochemical analysis. Following blood collection, the hippocampus was removed and placed in pre-cooled Eppendorf containers and snap-frozen in liquid nitrogen before storage in a Bio Ultra freezer (Snijers Scientific, Tilburg, Netherlands) at − 80 °C.

## Biochemical analysis

Plasma insulin, adrenocorticotropic hormone (ACTH) and corticosterone (CORT) concentrations were measured using their respective rat competitive-ELISA kits (Elabscience Biotechnology Co., Ltd, Wuhan, China) according to the manufacturer’s instructions.

## Real-time quantitative PCR (RT-qPCR)

The harvested hippocampus tissue was subjected to RNA extraction using a ReliaPrep miRNA Cell and Tissue Miniprep System (Promega, USA). The purity and concentration of RNA were determined by Nanodrop 2000 (Thermo Scientific, Roche, South Africa). A purity ratio (A260/A280) of 1.7–2.1 was considered acceptable for conversion to cDNA. Total RNA (1 μg) was reverse-transcribed into cDNA using a GoTaq® 2-Step RT-qPCR System as a cDNA synthesis kit (Promega, USA) following the manufacturer’s instructions. The BIO-RAD iTaq Universal SYBR Green I master mix was used to perform the PCR amplification on ROCHE LightCycler96 (Roche, South Africa). Primer sequences (Metabion, Germany) used in this study can be found in Table 1 below. PCR was performed using the following cycling conditions: Pre-incubation for 10 min at 95 °C, followed by 45 cycles of 95 °C for 15 s, 60 °C for 30 s and 72 °C for 30 s. The RT-qPCR results were analysed using the 2^−ΔΔCq^ comparative method relative to the control groups [35]. Glyceraldehyde-3-phosphate dehydrogenase (GAPDH) was used as the house keeping gene.

**Table 1:** List of primers used in the study

Sequence name	Sequence
Nr3c1 gene (glucocorticoid receptor)	Forward: 5′-ACCTCGATGACCAAATGACC-3′ Reverse: 5′-AGCAAAGCAGAGCAGGTTTC-3′
Nr3c2 gene (mineralocorticoid receptor)	Forward: 5′-AAAGGGTAGTGTGTGCAGGG-3′ Reverse: 5′-GTTCTCCTAGTTCCCGGAGG-3′
GAPDH	Forward: 5′-AGTGCCAGCCTCGTCTCATA-3′ Reverse: 5′-GATGGTGATGGGTTTCCCGT-3′

### Statistical analysis

All data were expressed as means ± S.E.M. Statistical comparisons were performed with Graph Pad InStat Software (version 5.00, Graph Pad Software, Inc., San Diego, California, USA) using student t-test for phase 1 of the study. One-way ANOVA was used for phase 2 of the study, followed by the Bonferroni post hoc test, which was used for the analysis of differences between control and the experimental groups. A value of *p* < 0.05 was considered statistically significant.

## Results

### HOMA-IR, HOMA-S and HOMA-β indices, plasma adrenocorticotrophic hormone (ACTH) and corticosterone (week 20)

At the end of 20 weeks, glucose handling by assessing insulin sensitivity and beta-function cell through the HOMA-IR, HOMA-S and HOMA-β indices were measured along with plasma ACTH and corticosterone concentration. The results in Table 2 showed that HOMA-IR value for NPD was within the insulin-sensitive range (< 1.0) while the PD group had a significantly higher HOMA-IR value compared to the PD which was in the range of significant insulin resistance. HOMA-S percentage of the PD was significantly lower than the NPD group while the HOMA-β percentage of the PD group was significantly higher in comparison to the NPD group. The results also showed that the PD group had similar ACTH concentration compared to the NPD group. However, the PD group had a significantly higher corticosterone concentration compared to the NPD.

**Table 2:** HOMA-IR, HOMA-S and HOMA-β indices, ACTH and corticosterone plasma concentrations in non-pre-diabetic (NPD) and pre-diabetic (PD) rats (n = 6, per group)

Groups (n = 6)	Plasma glucose (mmol/L)	Plasma insulin (pmol/L)	HOMA-IR values	HOMA-S values (%)	HOMA-β values (%)	Plasma ACTH (pg/ml)	Plasma corticosterone (ng/ml)
NPD	4.60 ± 0.45	30.42 ± 3.26	0.56	178.5	77,1	731.00 ± 23.22	526.20 ± 3.89
PD	6.08 ± 0.05*	322.80 ± 17.56	5.92 ***	16.9	226,6	747.30 ± 9.60	574.40 ± 15.77 *

Values are expressed as mean ± SEM

*p < 0.05; ***p < 0.001 denotes comparison with NPD

### HOMA-IR, HOMA-S and HOMA-β indices, plasma ACTH and corticosterone (week 32)

At the end of 32 weeks, glucose handling by assessing insulin sensitivity and beta-cell function through the HOMA-IR, HOMA-S and HOMA-β indices were measured along with plasma ACTH and corticosterone concentration. Table 3 shows the HOMA-IR index assessment for glucose handling in PD and NPD with measurements of plasma ACTH and corticosterone (CORT) concentration measured in non-stressed non-prediabetic (NPD) and prediabetic (PD) rat at week 32. HOMA-IR value for NPD was within the insulin-sensitive range (< 1.0) while the PD group had a significantly higher HOMA-IR value compared to the PD which was in the range of significant insulin resistance. HOMA-S percentage was significantly lower in the PD group in comparison to the NPD group while the HOMA-β percentage of the PD group was significantly higher compared to the NPD group. The results further showed that there was a decrease in plasma ACTH concentration in PD group compared to the NPD. CORT in the PD group was slightly increased compared to the NPD.

**Table 3:** HOMA-IR, HOMA-S and HOMA-β indices, ACTH and corticosterone plasma concentrations in non-pre-diabetic (NPD) and pre-diabetic (PD) rats (n = 6, per group)

Groups (n = 6)	Plasma glucose (mmol/L)	Plasma insulin (pmol/L)	HOMA-IR values	HOMA-S values (%)	HOMA-β values (%)	Plasma ACTH (pg/ml)	Plasma corticosterone (ng/ml)
NPD	5.18 ± 0.36	22.43 ± 3.98	0.43	234.2	49.4	706.50 ± 11.35	147.20 ± 1.37
PD	5.80 ± 0.44	145.30 ± 4.73	2.75*	36.3	141.2	635.80 ± 13.84 **	157.40 ± 1.70**

Values are expressed mean ± SEM

*p < 0.05; **p < 0.01, denotes comparison with NPD

### Stressed animals: forced swim test, plasma ACTH and corticosterone

**Fig. 1:**
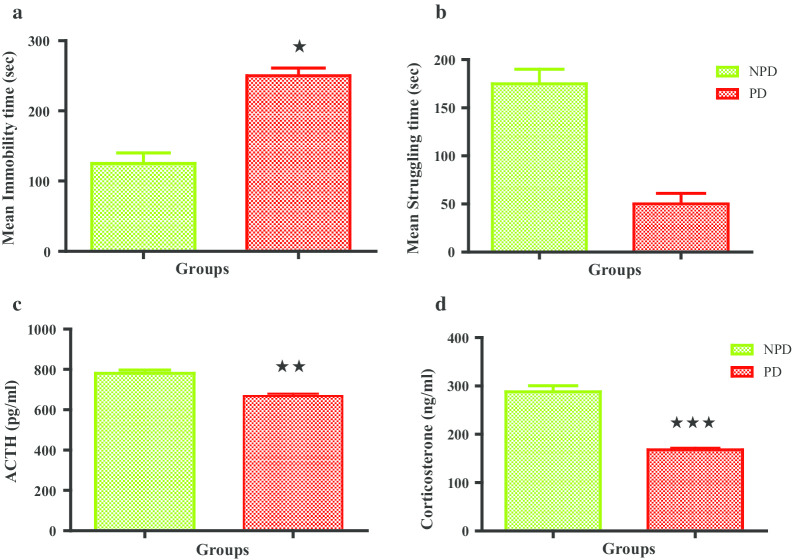
Mean immobility time (**a**) and mean struggle time (**b**) in stressed non prediabetic (NPD) and prediabetic (PD) rats (n = 6, per group). ACTH (**c**) and Corticosterone (**d**) plasma concentrations in stressed non prediabetic (NPD) and prediabetic (PD) rats (n = 6, per group). Values are expressed as mean ± SEM. *p < 0.05; **p < 0.01; ***p < 0.001 denotes comparison with NPD

Figure 1 displays the mean immobility time and mean struggle time forced swim test as well as the plasma ACTH and corticosterone concentrations for the stressed non-prediabetic and prediabetic groups. The PD group experienced a significantly increased immobility time compared to the NPD while the PD group showed a significantly decreased struggle time compared to NPD group. ACTH concentration of the stressed PD group was significantly decreased compared to the stressed NPD. The stressed PD group had a substantially lower concentration of corticosterone than the stressed NPD group.

### Hippocampal glucocorticoid and mineralocorticoid receptors

**Fig. 2:**
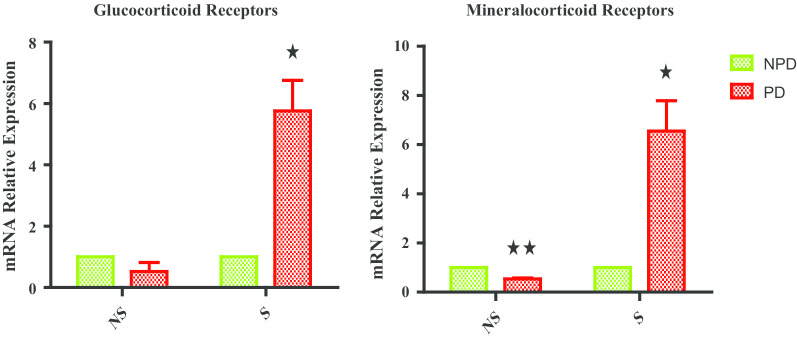
Glucocorticoid and Mineralocorticoid receptors in non-stressed (NS) and stressed (S) non-prediabetic (NPD) and prediabetic (PD) rats (n = 6, per group). Values are expressed as mean ± SEM. *p < 0.05; **p < 0.01 denotes comparison with NPD

Figure 2 shows hippocampal glucocorticoid (GR) and mineralocorticoid receptor (MR) gene expression measured in non-stressed (NS) and stressed (S) non-prediabetic and prediabetic group at the end of week 32. The NS PD group experienced a half-fold decrease in both GR and MR gene expression relative to the NS NPD group. The S PD group experienced a four-fold increase in GR gene expression while there was a seven-fold increase in MR gene expression relative to the NPD group.

## Discussion

A diet high in fats and carbohydrates has been identified as a factor in the increased prevalence of T2DM [26, 36]. However, studies indicate that complications often associated with T2DM begin during pre-diabetes [7]. Previous studies done in our laboratory have found that micro- and macrovascular changes leading to complications such as cardiovascular complications and non-alcoholic fatty liver disease seen in T2DM have been shown to begin during a prediabetic state [37, 38]. Hence, this current study was aimed at investigating the changes in the functioning of the HPA axis in a HFHC diet-induced prediabetic rat model.

Pre-diabetes has been reported to be associated with elevated blood glucose concentration (2). The findings of the present study coincided with a recent study conducted in our laboratory that reported that prolonged exposure to a HFHC diet supplemented with 15% fructose in rats resulted in elevated blood glucose concentration and a disturbance in glucose tolerance [29]. The current study confirms that ingesting the HFHC diet results in the increase in blood glucose concentrations which was seen during the fasting plasma glucose concentration resulting in impaired fasting glucose. This suggests that glucose utilization in insulin-dependent peripheral tissues such as skeletal muscles is decreased [39]. Glucose is increased by the breakdown of dietary carbohydrates, thus promoting insulin secretion and increasing insulin in circulation for glucose uptake by the tissues cells [40]. However, sufficient glucose is not taken up by the peripheral tissues suggesting there is some insulin resistance from the tissues as seen by the elevated glucose levels in plasma [40]. The insulin resistance indicated by the HOMA-IR index in the results could be due to the high intake of dietary fat, which increases the triglycerides due to decrease in expression of apo-B resulting in accumulation of LDLs [41]. As insulin is released under normal regulated physiology, the peripheral tissues are exposed to the free fatty acid (FFA) which induces insulin resistance due to the sufficient amount of insulin not producing adequate insulin response resulting in pancreatic β-cells producing more insulin as observed in the elevated plasma insulin and HOMA-β index [42]. This is to compensate for the high blood glucose concentrations that are found among the insulin resistant peripheral tissues [43].

Elevated plasma glucose and plasma insulin concentration though seen in a prediabetic state, have been found to have similar results in type 2 diabetic patients with a heightened HPA axis activity. Glucocorticoid (GC), cortisol in humans and corticosterone (CORT) in rodents, has been suggested to be a possible link in insulin resistance seen in elevated blood glucose concentration and imbalance of lipids as seen in previous studies [37]. We evaluated the HPA axis activity by measuring two components of the HPA axis under basal non-stressful conditions and found plasma ACTH levels did not change significantly, whereas CORT concentration increased in a prediabetic state. Various studies which studied the HPA axis functioning in type 2 diabetic patients have given conflicting results. Several studies found that HPA axis activity is increased [44, 45], whereas a different study found that the HPA axis is not heightened in diabetic individuals without diabetic complications [44]. However, individuals with severe diabetic complications showed increased HPA axis activity [44]. These results coincided with a study that showed that ACTH concentration did not change in diabetic individuals, but the cortisol concentration was increased [44]. The same study associated this phenomenon to impaired feedback mechanism of the HPA axis [44]. The current study’s results suggest that CORT concentration in a prediabetic state in the rat model had increased to a new basal secretion concentration to compensate for the increase in blood glucose concentration [45]. It would also suggest that the negative feedback mechanism was impaired as the CORT concentration did not decrease in response to the ACTH concentration [45]. Another possible contributor to the increased baseline secretion concentration of CORT is fructose [46]. High fructose intake had shown to heighten the HPA axis, increasing the baseline cortisol secretion [46]. Though the increase in glucose and lipids can also be attributed to the dietary intake, the increase in CORT concentration which has been shown to induce insulin resistance is a possible attributor of the increase in glucose levels and the derangement of lipids.

Elevated glucocorticoid concentration not only leads to insulin resistance, but it has been shown that increased glucocorticoids (GCs) decrease insulin production, secretion and sensitivity [47–49]. The increase in GCs, particularly during stress, is designed to increase energy availability by increasing glucose output from the liver while simultaneously disturbing insulin action [11, 49, 50]. However, in an environment where elevated GC exposure is prolonged even under non-stressful conditions, as shown in our results, this environment may cause increased interference of insulin action [11, 50]. Glucocorticoids have been shown to inhibit the pancreatic-β cells from secreting insulin directly, impair insulin-mediated glucose uptake and interfere in the insulin signalling cascade in peripheral tissues such as skeletal muscles [47, 48, 51]. Studies have shown that a compensatory mechanism is activated in healthy individuals during acute glucocorticoid-insulin resistance where pancreatic-β cells increase their function or insulin release [49, 52]. However, in individuals or rodents where insulin sensitivity is decreased, and insulin resistance is increased such as the prediabetic animal model in this study, the compensatory mechanisms counteract the prolonged glucocorticoid-induced insulin resistance resulting in hyperglycaemia [49, 52–54]. Hyperglycaemia can also be attributed to glucocorticoids function in regulating glycogen reserves in the liver by promoting gluconeogenesis, thus increasing glucose levels for energy production whereby elevated glucocorticoid upregulates its function [12, 55]. The further increase of glucocorticoids in a stressful event could then trigger exacerbated hyperglycaemia, increasing the risk of depression.

The distress of a new T2DM diagnosis, managing T2DM as well as the addition of everyday increase of unpredictable stressors have been shown to contribute and exacerbate complications seen in T2DM patients [56, 57]. Furthermore, the increase in stress has been linked to diabetic patients presenting with depressive symptoms and showing an increased risk of depression [58]. Depression is a complex heterogeneous neurological disorder where one of the main physiological manifestations is the dysregulation of the HPA axis as a result of chronic stress [59]. It has been shown that the combination of chronic stress and T2DM can cause further dysregulation of the HPA axis [9, 60]. This is further worsened by a sedentary lifestyle and excessive consumption of high caloric diet which often refined carbohydrates and saturated fats [61, 62]. The induction of pre-diabetes for 20 weeks was associated with elevated corticosterone concentrations at under non-stressful conditions [34]. So, we further investigated the behavioural and physiological response of diet-induced prediabetic rats with the addition of mild chronic stress.

Behavioural changes are typically the first signs or symptoms of individuals who are depressed [63]. In an inescapable environment, with the individual having experienced chronic stress prior, increased psychomotor retardation has been shown to be a symptom of depression [64]. This study looked at the psychomotor response of chronically stressed-induced prediabetic and non-prediabetic rats using the forced swim test, a behaviour assay. This behaviour test is performed to assess the psychomotor responses by placing the individual rodents in an inescapable stressful environment to see how they respond [65]. The use of the forced swim test as an assay for behavioural observation of depression has been a source of scientific controversy over the years [66, 67]. The immobility of the rodents has been suggested to be an adaptive behaviour rather than a representation of an internal state of defeat leading to psychomotor retardation as seen in humans [66, 68]. While this is a possibility, there are various studies which have shown the use of this test to be viable in showing the individual rat’s coping response to stress [67, 69, 70]. Multiple studies that have investigated antidepressants have shown that the induction of chronic stress in rodents has resulted in the state of defeat which has been likened to psychomotor retardation in humans with depression [67, 69, 70]. The use of some of these antidepressants was shown to reduce the immobility of the once immobile rats after treatment by seeing an increase in struggling time [67, 69, 70]. Immobility of the rat, known as a state of defeat, includes very minute movements to remain above water to breathe without the vigorous struggle of trying to escape as seen in the non-prediabetic group with increased struggle time [71]. The prediabetic group experienced an increased overall immobility time. The associated psychomotor retardation to increased immobility may be attributed to the chronic stress induction received a few days prior, which may have resulted in increased corticosterone [72, 73]. Chronic stress has been shown to be associated with depressive symptoms due to the constant activation of the HPA axis, which increases GC concentration in circulation [74]. The continual increase in glucocorticoids travels to the brain where constant, elevated GCs in the highly regulated brain may cause dysregulation of receptors in the hippocampus which may bring about dysfunction of the HPA axis resulting in the behavioural changes seen [75]. The increase in psychomotor retardation can also be attributed to the high caloric intake, which included a high intake of fat and fructose. It has been shown that increased consumption in fructose has been linked to increased risk of depressive-like symptoms where elevated fructose in circulation has been shown to accumulate in the brain and result in dysregulation of the reward centres found in the limbic system in the brain contributing to the symptoms seen in depressive individuals [76].

The HPA axis is a tightly regulated pathway which plays a vital role in the stress response [77]. However, T2DM has been shown to cause dysregulation of the HPA axis resulting in the increased risk of depression [58]. One of the physiological manifestations seen in individuals with depression coupled with T2DM is the dysregulation of the two major components of the HPA axis, ACTH as well as GC which is cortisol in humans and corticosterone (CORT) in rodents [44, 58]. At the initial state of pre-diabetes at week 20, the results showed that ACTH basal concentration in non-stressful conditions did not have significant change, however, once the prediabetic state was prolonged there was a decrease in ACTH concentration in the same non-stressful conditions. Corticosterone concentrations remained consistently high even as the prediabetic state was prolonged in the non-stressful condition. Ideally, a decrease in ACTH should correlate to CORT concentration, and CORT concentration should decrease as a form of negative feedback [77]. However, the differences in concentrations of the two hormones could be an indication that the prolonged prediabetic state may cause impaired negative feedback [44]. In addition, signalling between the ACTH and adrenal gland may have also been impaired as an increase in corticosterone under basal non-stressful condition could be a result of the high caloric diet [78]. A study showed that a diet high in fat resulted in hyperplasia of the adrenal cortex and increased expression of multiple genes involved in steroidogenesis, including the production of CORT [78]. Several studies, however, have reported that fructose is able to pass the blood–brain barrier (BBB) via the glucose transporter 5 (GLUT 5) which has a high affinity to fructose and shown to be found on the BBB as well as areas in the brain such as the hypothalamus and hippocampus [79, 80]. Fructose found in excess in the peripheral circulation from increased fructose intake passes the BBB and accumulates in the hypothalamus, which can result in toxicity leading to activation of the HPA axis [46, 80]. Our study also looked at these two components after chronic stress was induced and found that prediabetic stressed animals had ACTH concentration that followed the same trend as prediabetic non-stressed. The CORT concentrations in the prediabetic stressed animals experienced a significantly decreased concentration which may reiterate an indication of impaired signalling and feedback [44]. This may have been due to insufficient ACTH, which was not able to stimulate the adrenal cortex resulting in a decrease in CORT [81]. In addition, this could also be a result of adrenal fatigue as it has been reported that the adrenal gland may experience hypertrophy due to a high fat diet and chronic stress which could increase secretion of the hormones [78, 82]. However, in an adverse situation such as chronic stress, this could result in exhaustion of the adrenal gland in secreting these hormones, including the CORT [78, 82]. The diet-induced prediabetic state especially one where the diet is high in saturated fats and refined carbohydrates may dampen the ACTH release causing decreased activation of the HPA axis during the stress [83, 84]. The CORT concentrations of the prediabetic stressed animals correlates to the assumption that highly palatable food like the HFHC diet given to the prediabetic animals may have dampen the stress response [83, 84]. Moreover, when we observe the previous behaviour results and receptors involved in stress response management and correlate the ACTH levels to CORT concentrations the supposition would be that CORT decreased after consumption of the palatable HFHC diet following the stress. It has been shown that palatable westernized food can dampen the stress response by activating reward centres in the limbic area of the brain resulting in the decrease in HPA axis activation during stress [50, 76].

Glucocorticoids (GCs) are mediated by two receptors, mineralocorticoid receptors (MR) and glucocorticoid receptors (GR) [85]. These receptors are found in various tissues in the body, including the brain where binding of GCs to these receptors mediate various physiological responses, including the stress response [85]. MRs expression is limited in the brain and can be found in the limbic areas of the brain such as the hippocampus and amygdala playing a role in the early response to stress, whereas GRs are expressed through the brain [86]. In the hippocampus, GR’s function is mediating a negative feedback response to the hypothalamus [87]. In a state of rest, GCs binds more to MRs as MRs have a tenfold higher affinity for GCs than GR in the hippocampus [75]. However, during a stress response, GCs are increased resulting in the increased expression of GR’s while MR expression becomes decreased which can be seen in the results of the receptor gene expression and CORT concentrations of the control non-stressed groups [75]. Increased expression of GRs in the hippocampus during stress results in increased GR-CORT binding, which triggers a negative feedback regulation where GR-CORT ligand complex initiates signalling to the hypothalamus, causing a decrease in CRH secretion [88]. A decrease in CRH results in a decrease of ACTH secretion in the anterior pituitary gland leading to a decrease in glucocorticoids secretion in the adrenal gland and eventually restoring the body back to its non-stressed physiological condition [77, 89]. However, persistent activation of the HPA axis due to chronic stress results in the HPA axis being hyper-activated causing an abnormal increase of GCs [75, 89]. The subsequent abnormal increase causes downregulation of GRs in the hippocampus and an upregulation of MRs, which has been associated with depressive symptoms [90]. Increased MR expression during stress as seen with the prediabetic stressed group has been shown to decrease CRH inhibition, causing impaired feedback which could explain the behaviour modifications seen in depressed rodents. A novel pathway was described by Zhou et al., as a possible mechanism in the downregulation of impairment of GRs and upregulation of MRs as one of the aetiologies in depression. MR binding to corticosterone results in MR activation, causing an upregulation in nNOS expression increasing nitric oxide (NO). Increased NO interrupts and interacts with the sGC-cGMP and hippocampal MAPK pathways respectively resulting in the downregulation of GR expression. This causes decreased inhibition of the hypothalamus resulting in further secretion of CRH leading to the hyperactivation of the HPA axis and subsequent behaviour change seen in the prediabetic stressed group [91]. However, the increase in GR expression at gene level could be attributed to fructose from the diet. It has been reported that increase fructose in the hippocampus along with other areas in the brain, specifically the limbic areas responsible for reward and pleasure, increase GR expression [50, 76]. The upregulation is a result of the activation of reward centres in the brain where palatable food which includes fructose has been shown to dampen the stress response by inhibiting the HPA axis activity during a stress response [76, 83].

The study had a few limitations such as the need to analyse lipid profile to further confirm the impact of GCs and diet in causing hyperlipidemia and dyslipidemia. Additional behavioural studies such as the use of the elevated plus maze used in analysing anxiety symptoms would have been beneficial in further understanding the association between prediabetes and the consequence of a stressed activated HPA axis. Further investigation of other components of the stress response such as catecholamines and cytokines involved in fight, flight or freeze response to stress would have been beneficial. However, the collective results obtained in this study warrants the need to look at this association in humans which can further drive the need for more research in preventative measures against T2DM in the prediabetic state and further improve the diagnostic methods of prediabetes in order to prevent the progression of T2DM as this state is reversible. The evidence in this study also warrants the need to investigate mental health deterioration in association with prediabetes and how diet contributes to both states.

## Conclusion

In conclusion, HFHC diet-induced prediabetic state is associated with dysregulation of the HPA axis was evidenced by the elevated basal CORT concentration along with the unchanged ACTH concentration in non-stressed conditions. In addition, the behavioural response is affected by the prediabetic state in combination with chronic stress which was evidenced by increased immobility time liken to psychomotor retardation as well as the elevated receptors altering the stress response. Collectively, these observations not only suggest that high caloric diet-induced pre-diabetes increases the risk of progression of pre-diabetes to T2DM, but pre-diabetes may predispose individuals to an additional diagnose of depression if interventions to reverse pre-diabetes are not taken.

## Data Availability

The datasets generated during the current study are available from the corresponding author on reasonable request.

## Abbreviations

ACTH: Adrenocorticotropic hormone
ADA: American Diabetes Association
ANS: Autonomic nervous system
BBB: Blood brain barrier
BRU: Biomedical Research Unit
CORT: Corticosterone
CRH: Corticotropic releasing hormone
ELISA: Enzyme linked immunosorbent assay.
FFA: Free fatty acid
FST: Forced swim test
GC: Glucocorticoids
GLUT: Glucose transporter
GR: Glucocorticoid receptor
HFHC: High fat high carbohydrate
HPA: Hypothalamic–adrenal–pituitary
IDF: International Diabetes Federation
IFG: Impaired fasting glucose
IGT: Impaired glucose tolerance
MR: Mineralocorticoid receptor
nNOS: Neuronal nitric oxide synthase
NO: Nitric oxide
NPD: Non-prediabetes
OGTT: Oral glucose tolerance test
ONOO: Peroxynitrite
PD: Prediabetes
PVN: Paraventricular nucleus
T2DM: Type 2 diabetes mellitus
UCMS: Unpredictable chronic mild stress
UKZN: University of KwaZulu-Natal
WHO: World Health Organisation
